# Social deficits in BTBR T+ Itpr3tf/J mice vary with ecological validity of the test

**DOI:** 10.1111/gbb.12814

**Published:** 2022-05-27

**Authors:** Maciej Winiarski, Ludwika Kondrakiewicz, Kacper Kondrakiewicz, Joanna Jędrzejewska‐Szmek, Krzysztof Turzyński, Ewelina Knapska, Ksenia Meyza

**Affiliations:** ^1^ Laboratory of Emotions Neurobiology, BRAINCITY – Center of Excellence for Neural Plasticity and Brain Disorders, Nencki Institute of Experimental Biology Polish Academy of Sciences Warsaw Poland; ^2^ NeuroElectronics Research Flanders Leuven Belgium; ^3^ Laboratory of Neuroinformatics, Nencki Institute of Experimental Biology Polish Academy of Sciences Warsaw Poland; ^4^ Faculty of Physics University of Warsaw Warsaw Poland

**Keywords:** autism, BTBR T+ Itpr3tf/J, Eco‐HAB, social affiliation, social network dynamics, validation

## Abstract

Translational value of mouse models of neuropsychiatric disorders depends heavily on the accuracy with which they replicate symptoms observed in the human population. In mouse models of autism spectrum disorder (ASD) these include, among others, social affiliation, and communication deficits as well as impairments in understanding and perception of others. Most studies addressing these issues in the BTBR T+ Itpr3tf/J mouse, an idiopathic model of ASD, were based on short dyadic interactions of often non‐familiar partners placed in a novel environment. In such stressful and variable conditions, the reproducibility of the phenotype was low. Here, we compared physical conditions and the degree of habituation of mice at the time of testing in the three chambered social affiliation task, as well as parameters used to measure social deficits and found that both the level of stress and human bias profoundly affect the results of the test. To minimize these effects, we tested social preference and network dynamics in mice group‐housed in the Eco‐HAB system. This automated recording allowed for long‐lasting monitoring of differences in social repertoire (including interest in social stimuli) in BTBR T+ Itpr3tf/J and normosocial c57BL/6J mice. With these observations we further validate the BTBR T+ Itpr3tf/J mouse as a model for ASD, but at the same time emphasize the need for more ecological testing of social behavior within all constructs of the Systems for Social Processes domain (as defined by the Research Domain Criteria framework).

## Graphical abstract

The data shown here challenges the classical view that social deficits in mouse models of neurodevelopmental disorders should be adressed with brief, dyadic tests of social affiliation. They encourage taking a more ethological approach, employing group testing of animals in semi‐natural environments over periods of multiple days. Such testing conditions are not only less stressful for the animal, but also permit unfolding of a full repertoire of social behaviors, which in turn allows for a more thorough search for impairments across multiple constructs of the Systems for Social Processes Domain of the RDoC.

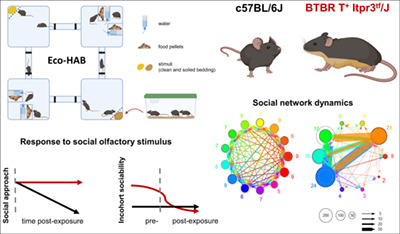

## 1 INTRODUCTION

Social deficits observed in many neurodevelopmental disorders encompass a wide spectrum of symptoms including impaired social affiliation, perception and understanding of others, and communication. Each of these constitutes a separate construct within the Systems for Social Processes Domain of the Research Domain and Criteria (RDoC) framework. To address these deficits and understand the neuronal mechanisms involved in their development adequate animal models and behavioral testing strategies are required. Constant validation of animal models is necessary because of genetic drift and changes in the immune status of animals between facilities, which can readily affect the outcome of behavioral testing. Genetic drift has been a huge concern for inbred mouse strains and certain mutant lines, which with time may lose their asocial phenotype.^1^, ^2^, ^3^ Stable immune profile on the other hand is important in mouse strains repeatedly showing pro‐inflammatory systemic and brain phenotype, for example, the most popular idiopathic mouse model of autism spectrum disorder (ASD), the BTBR T+ Itpr3tf/J mouse (BTBR).^4^, ^5^, ^6^, ^7^ In this strain, changes to the immune status have been shown to either improve sociability^8^, ^9^, ^10^ or aggravate the deficit,^11^ depending on the way the manipulation affected the immune system of the subjects.

Most studies testing sociability in mouse models of ASD employ brief tests of social affiliation, during which subject mice are placed in a novel environment by an unfamiliar Experimenter.^12^ The interaction is primarily dyadic in nature and often restricted by a mesh restrainer disabling physical contact between individuals. While the latter might reduce stress coming from an agonistic social encounter, it also profoundly limits the social repertoire of the animals. Testing conditions and their stressfulness are an important variable to be considered when planning tests of sociability as neuronal circuits for fear and anxiety are intertwined with those regulating social motivation (for review see References 13, 14). Human intervention during the experiment (necessary for protocols such as the social affiliation tests in the three chambered apparatus) may also provide a source of unnecessary stress,^15^ especially in animals not habituated to interaction with the Experimenter. Previous studies point to disruptive effect of stress on sociability,^16^, ^17^ and to the potential for social interactions to become a source of stress,^18^ especially in environments not permitting escape. All that data points to the necessity of using more ethologically inspired approach when investigating sociability deficits in mice. Here we provide support for this notion by showing that social affiliation tests run under conditions varying with stressfulness (light intensity, habituation level and previous exposure to enriched environment) give different results. We then put these results in perspective of the data obtained in the semi‐natural environment of the Eco‐HAB system.^19^

Until recently analysis of such ethologically relevant, longitudinal tests (for example in the Visible Burrow System, for review see References 20, 21) was either very labor intensive^22^ and/or required subsampling^23^ of data. In recent years, improvements have been made to both individual animal recognition in long‐term video recordings and in other forms of tracking of animal activity. Systems based on image recognition employed either color coding,^24^ tag recognition^25^, ^26^ or were combined with another tracking method (e.g., with radio frequency tagging, RFID^27^, ^28^, ^29^). The Eco‐HAB system offers an alternative approach eliminating the need for video recording by compartmentalization of the social arena.^19^, ^30^ Here we employed this approach and resolved to score as many behavioral parameters as possible to describe the social behavior of group housed c57BL/6J (B6) and BTBR mice.

To systematically examine the impact that testing conditions might have on social motivation in the standard (three chambered apparatus) social affiliation test, we compared cohorts of B6 and BTBR mice in radically different settings. On one side of the spectrum of stressfulness, we tested naïve animals in standard office (bright) light conditions (540 lux, a legally required luminosity for office spaces in Poland). On the other side (low stress), we tested animals habituated to both transportation and human handling and tested them in dim light (25 lux). To further characterize this effect in the BTBR strain we compared naïve mice, to mice exposed to either type of habituation described above or to enriched environment prior to testing.

The choice of parameters reported in social behavior studies is prone to human bias and should not be underestimated. Classical tests of social affiliation usually report time or distance traveled in “social” parts of an arena. Here we looked at five different parameters (scored both automatically and manually) to assess their validity for description of social affiliation and motivation (both falling under Social Affiliation and Attachment construct of the RDoC). We then aimed at verifying whether testing of voluntary group social interactions in the Eco‐HAB system would let us infer on other forms of social behavior, with main focus on social network stability and perception of social stimuli (falling under Perception and Understanding of Others and Social Communication Constructs of the RDoC). By combining information from both types of testing, we hoped to further validate the BTBR mouse strain as a mouse model of ASD and raise awareness of the benefits coming from testing multiple aspects of social behavior rather than a single construct with one unit of behavioral analysis.

## 2 MATERIALS AND METHODS

### 2.1 Animals and housing conditions

All experimental procedures were conducted in accordance with ethical standards of the European Union (directive no. 2010/63/UE) and Polish regulations and were pre‐approved by the Local Ethics Committee. C57BL/6J (B6) male mice were bred at the Animal House of Nencki Institute of Experimental Biology, Polish Academy of Sciences. BTBR T+ Itpr3tf/J (BTBR) breeding pairs (Stock No: 002282) were purchased to the from the Jackson Laboratory and bred at the Animal House of Nencki Institute of Experimental Biology, Polish Academy of Sciences. Male offspring of both lines was transferred from the breeding facility to rooms adjacent to the experimental room at 2–2.5 months of age (at least 2 weeks prior to the onset of any testing). Mice were group‐housed (typically in *n* = 10–13 non‐littermate groups, in standard plexiglass cages 56 cm × 34 cm × 20 cm with added nesting material and paper tubes) under 12 h/12 h light/dark cycle with water and food provided ad libitum. For experiments in the Eco‐HAB system, the onset of dark phase in the room was shifted to match the conditions used during the experiment (13:00–01:00 or 12:00–24:00, depending on daylight savings time). In all housing and experimental rooms, the temperature was maintained at 22–24°C, with humidity levels between 40% and 60%. Home cages were cleaned once a week. The number of animals used in a particular experiment is given in figure descriptions.

### 2.2 RFID tagging

For Eco‐HAB experiments mice were individually tagged (via subcutaneous injection under brief 5% isoflurane anesthesia) with sterile glass coated RFID microtransponders (9.5 mm − length and 2.2 mm − diameter, RFIP Ltd). After recovery from anesthesia mice were placed back with cagemates in the home cage with clean bedding. No apparent aggression was observed because of this manipulation.

### 2.3 Behavioral testing

#### 2.3.1 Social affiliation test in the three‐chambered apparatus

**FIGURE 1:**
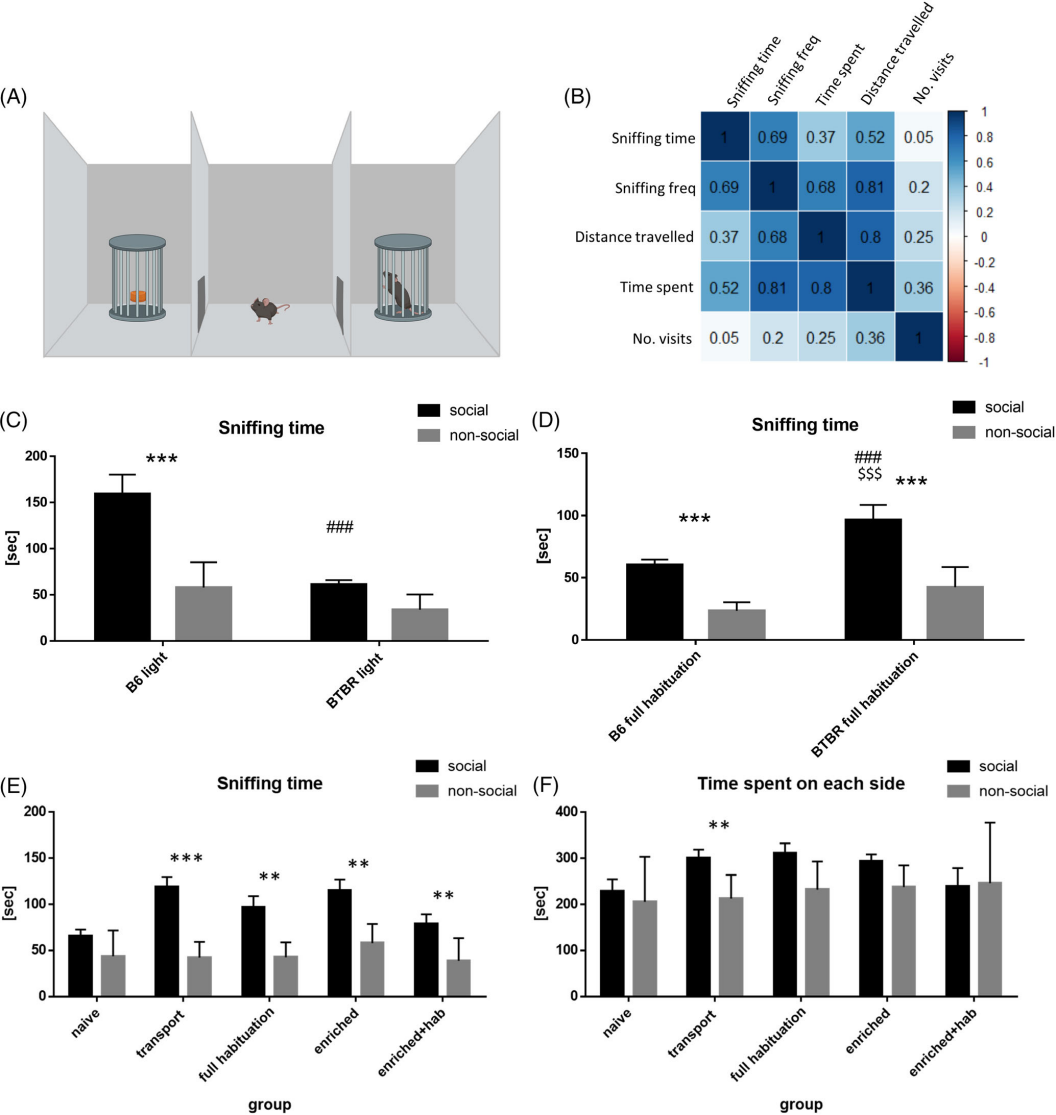
Social affiliation scores in the three chambered apparatus depend on stressfulness of testing conditions. (A) The schematic of the apparatus. (B) Correlation matrix for behavioral parameters scored manually (BehaView) and with the use of automated animal positioning software (EthoVision XT9) in all BTBR groups (*n* = 63). Values/colors represent *r*—Pearson correlation coefficients for pairwise comparisons. (C) Time spent by B6 (*n* = 10) and BTBR (*n* = 12) mice on sniffing social and non‐social stimuli in bright light conditions (540 lux). (D) Time spent by c57 (*n* = 12) and BTBR (*n* = 10) mice on sniffing social and non‐social stimuli in dim light conditions (25 lux) in groups submitted to habituation to both transportation and handling by the Experimenter. (E) The effect of behavioral manipulations on sniffing of stimuli by BTBR mice tested in dim light conditions (25 lux): naïve (*n* = 10), mice habituated to transportation alone (*n* = 12), mice habituated to both transportation and handling by the Experimenter (*n* = 10), mice previously living in the enriched environment of the Intellicage (TSE, DE) system (*n* = 10), mice previously living in the Intellicage (TSE, DE) system and then habituated to both transportation and handling by the Experimenter (*n* = 9). (F) The effect of behavioral manipulations on time spent on either side of the apparatus (social vs. non‐social) by BTBR mice (the same groups as in (E)). For (C)–(F), data are presented as mean ± SEM with black columns representing soc side and gray columns representing non‐soc side of the apparatus. For (C)–(D**)**, ANOVA was followed by Holm‐Sidak multiple comparison test, *** indicates *p* < 0.001 for within strain comparison (soc vs. non‐soc), ^###^ indicates *p* < 0.001 for between strain comparison (B6soc vs. BTBRsoc), ^$$$^ indicates *p* < 0.001 for between strain comparison (B6non‐soc vs. BTBRsoc). For (E)–(F), paired *t*‐test or Wilcoxon matched‐pairs signed‐rank test were used, ** indicates *p* < 0.01, *** indicates *p* < 0.001

Social affiliation test was performed using a gray three‐chambered apparatus, a 63 × 43 × 30 cm arena divided into three compartments (Figure 1A). The access to side chambers was limited with a set of retractable doors. The testing was performed in three consecutive sessions, each lasting 10 min. During the first session each experimental mouse was individually placed in the central chamber with no access to side chambers. After 10 min, the second session began with lifting the doors and allowing the access to side chambers equipped with an empty wire cup (DOKUMENT, currently available as https://www.ikea.com/gb/en/p/droenjoens-pen-cup-white-00494879/Ikea, SE, modified to weight it down) each. At the end of this phase the mouse was urged to return to the central chamber and the doors were closed. In one of the chambers (counterbalanced between trials) a stimulus mouse (an unfamiliar c57BL/6J male of similar age) was placed under the wire cup, while an inanimate object (a red polybutylene terephthalate PBT cap of a 1 L laboratory bottle, GL45, SCHOTT, DURAN) was placed under the cup in the other chamber. The doors were then removed, and the experimental mouse allowed to explore the arena freely. The arena, cups, and the inanimate object were washed with 70% ethanol between trials. The stimulus mice (*n* = 9) were habituated to being placed under cups for at least 3 days before the onset of the testing. The test was performed during the light phase of the LD cycle (lights on 08:00–20:00) in dim light condition (max. 25 lux at the arena floor), unless otherwise stated.

We have tested the mice in several conditions:

1. Naïve mice—mice did not undergo any habituation.
2. Bright light—naïve mice were tested in bright light (540 lux at the arena floor).
3. Transportation—for 5 days mice were transported to the experimental room and left there for 30 min undisturbed.
4. Full habituation—for 5 days mice were transported to the experimental room and then handled by the Experimenter. For the first 2 days the Experimenter inserted his/her hands into the home cage and did not attempt to touch the mice. On the following 3 days he/she would lift each mouse by the tail and place it gently on the palm of the hand. The mouse was permitted to jump back to the cage at will.
5. Enriched environment—mice previously housed for 2 weeks in the Intellicage system (TSE, DE) but did not undergo habituation to either transportation or the Experimenter.
6. Enriched environment and habituation—mice housed for 2 weeks in the Intellicage system (TSE, DE) and then exposed to full habituation (see condition number 4).

Each testing session was video recorded from above and analyzed offline with the use of both automated (EthoVision XT9, Noldus, NL) and manual (BehaView, Dr P. Boguszewski www.pmbogusz.net) tools.

Parameters scored included:

EthoVision:

- Distance traveled in each chamber (Tables [Link], [Link]).
- Time spent in each chamber (Figure 1F).
- Number of visits to each chamber (Tables [Link], [Link]).

BehaView:

- Active sniffing time near the cup (Figure 1C–E).
- Number of sniffing bouts (Tables [Link], [Link]).
- Number and duration of self‐grooming episodes (for characterization of non‐social behaviors of naïve BTBR mice).
- Number and duration of cage exploration bouts (for characterization of non‐social behaviors of naïve BTBR mice).

#### 2.3.2 The Eco‐HAB system

The Eco‐HAB system is a fully automated, RFID‐based system for testing spontaneous social behavior of groups of mice.^19^, ^30^ It consists of four polycarbonate cages (30 × 30 × 18 cm), two of which are equipped with food hoppers and water bottles. The two remaining cages are empty, and their outwards‐pointing corners are blocked off with a perforated partition (11.5 × 15 cm). All cage floors are covered with sterile wood‐chip bedding. The cages are connected with four transparent plexi tubes (30 cm long, 3.6 cm inner diameter, 4 cm outer diameter) equipped with 2 RFID antennas each (placed 5 cm away from the entrance on either side of the corridor). All housing elements can be autoclaved and disinfected with 70% alcohol. The data from RFID tag readings is saved every hour to a disc drive by custom made software. Python library for loading and analysis of RFID tag readings (PyEcoHAB) is open‐source: https://github.com/Neuroinflab/pyEcoHAB.

All behavioral tests employing the Eco‐HAB system were performed in duplicate.

##### Response to social stimulus

**FIGURE 2:**
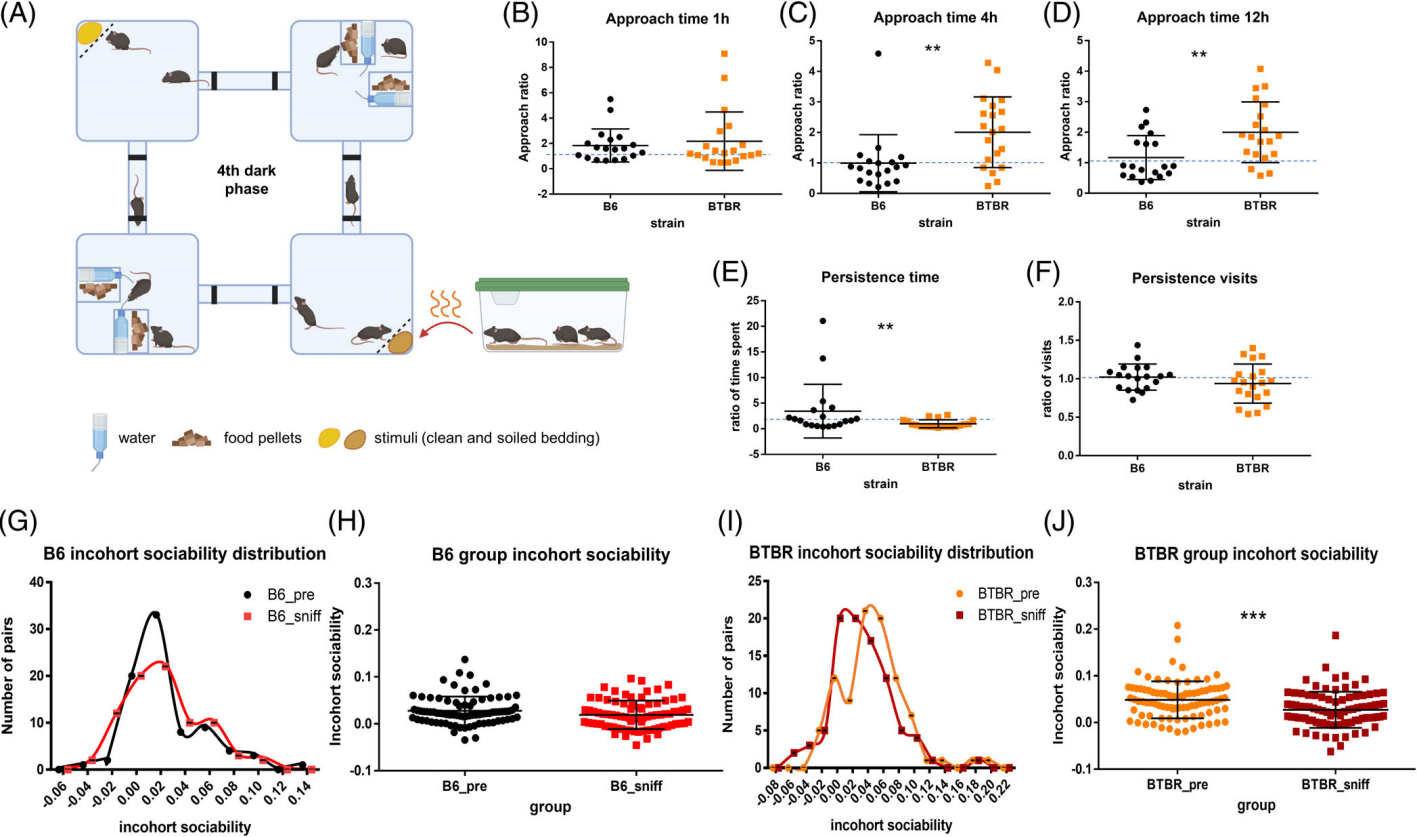
Difference in social affiliation displayed in response to scented bedding from a cage of unfamiliar B6 mice. (A) The schematic of the Eco‐HAB system on scent deposition day with marked bottle, feeder and bedding used as social stimulus (clean/control in yellow, soiled/social scent in brown). (B) Approach time during the first hour after deposition of the social scent. (C) Approach time during initial 4 h after deposition of the social scent. (D) Approach time during the entire dark phase following deposition of the social scent. (E) Persistence in the time spent in the compartment with the social stimulus. (F) Persistence in the number of visits to the compartment with the social stimulus. (G) Distribution of incohort sociability scores for B6 mice before and after presentation of social scent. (H) Lack of change in incohort sociability in B6 mice in response to the social scent. (I) Distribution of incohort sociability scores for BTBR mice before and after presentation of social scent. (J) Shift to lower values of incohort sociability in BTBR mice in response to the social scent. Group sizes for all comparisons: B6 *n* = 19, BTBR *n* = 20. Differences in (B)–(F) were assessed with either unpaired t‐test or U‐Mann Whitney test. ** indicates *p* < 0.01. Pairwise differences in incohort sociability (H and J) were assessed with Kolmogorow‐Smirnov test, *** indicates *p* < 0.001

This 4‐day paradigm consists of 3 days of habituation to the Eco‐HAB environment (Figure 2A), followed by exposure to social scent (soiled bedding from same sex, same age unfamiliar c57BL/6J mice) in one of the corners behind the partition. Control scent (clean bedding) is placed behind the partition in the opposite cage. Mice freely explore the cages throughout the experiment. The following parameters were recorded:

- changes in approach ratio (time spent in the chamber with social scent vs. control scent during the first dark phase after introduction of the stimulus compared with the same ratio from the last habituation day, here calculated for three time bins (1 h, 4 h, and 12 h) within the dark phase immediately following the introduction of the social stimulus, Figure 2B–D);
- persistence (proportion of visits and time spent in compartments (with social vs. control scent) was recorded during second half of the testing phase (as described above), divided by the same proportion from the first half of that phase, Figure 2E–F);
- incohort sociability (the excess amount of time any given pair of mice spent together above the amount of time they would spend together assuming independent exploration of the apparatus (measure described in detail previously,^19^, ^30^ Figure 2G–J). This parameter measures the propensity of each pair of mice within the cohort to voluntarily spend time together (other than by chance resulting from independent commuting between compartments of the arena) and as such captures social affiliation between members of such pairs.

##### Longitudinal observation of social network dynamics in the Eco‐HAB system

**FIGURE 3:**
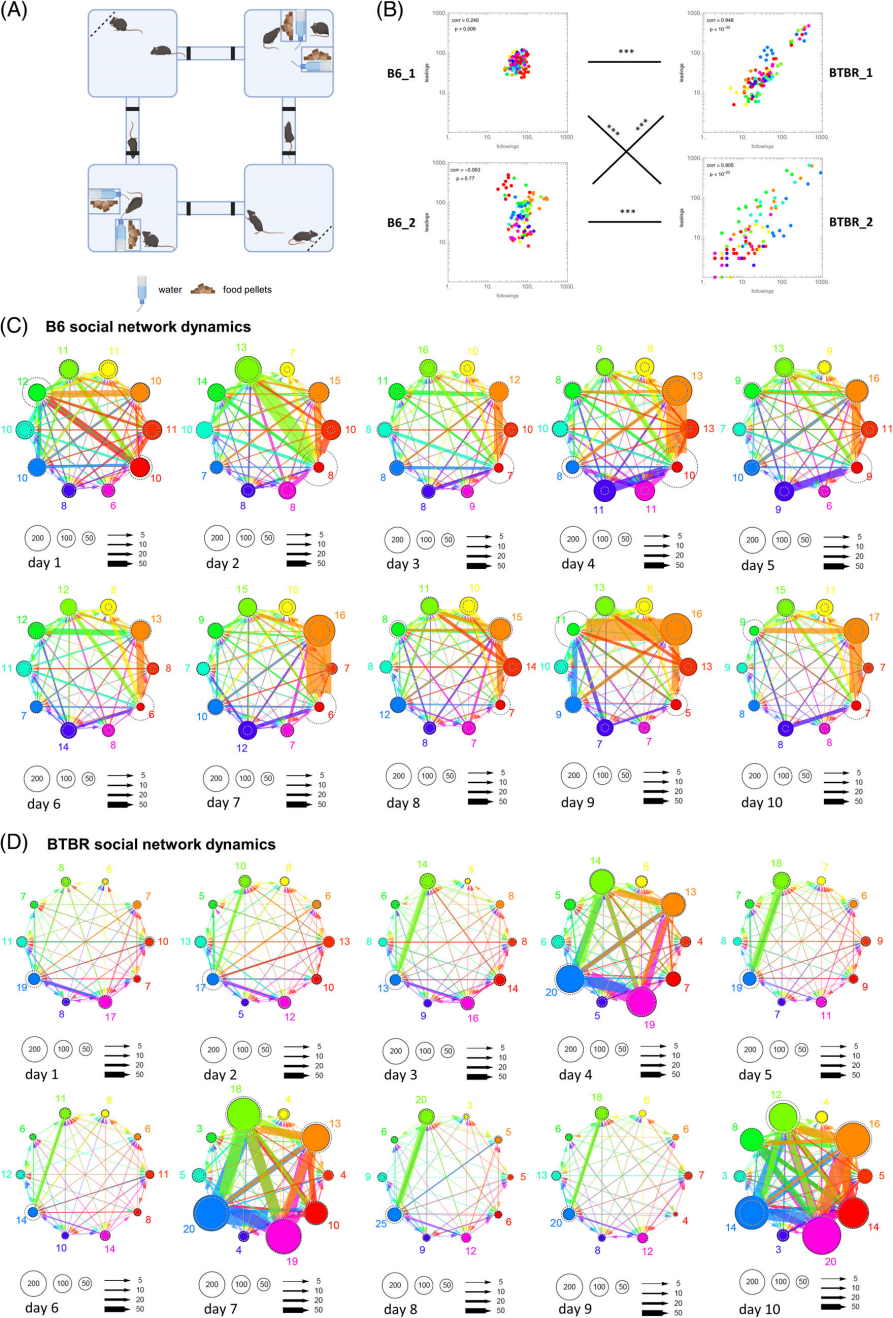
Social network dynamics is different in B6 and BTBR mice. (A) The schematic of the Eco‐HAB. The activity of mice was recorded for 10 days with no intervention or scent deposition. The bottle and feeder are indicated with symbols described in the legend. (B) Correlation of following and leading episodes measures for 10 consecutive days in four cohorts (B6_1 *n* = 13, B6_2 *n* = 10, BTBR_1 *n* = 10 and BTBR_2 *n* = 10). Pearson correlation coefficient and *p* values are given for each graph. The differences in the distribution of ranks between cohorts were assessed with Spearman correlation coefficient. (C) Visualization of social network dynamics in B6 cohort no. 2 (*n* = 10) over 10 consecutive days. (D) Visualization of social network dynamics in BTBR cohort no. 1 (*n* = 10) over 10 consecutive days. Graphs in (C)–(D) depict the strength of interaction between pairs of animals in each cohort: the size of the solid circle represents the number of times a mouse followed other mice, the size of the dashed circle represents the number of times the given mouse was followed by another individual. The thickness of arrows connecting pairs of mice represents the strength of their interaction. The color‐matched numbers by each node represent PageRanks (shown as %), that is, weights of nodes in directed following graphs.

To observe social network dynamics in group housed c57BL/6 and BTBR T+ Itpr3tf/J we tested their voluntary behavior in the Eco‐HAB system over the period of 10 days without any additional changes to the testing environment (Figure 3A). During this time, we recorded the activity of the animals with special focus on episodes of following. Followings (and leadings) were recorded for a pair of mice when two animals entered a tube connecting two cages one after another (the second animal entered the tube while the first was still in it) and left the tube in the same order and in the same direction. This measure was recorded as a “leading” for the first animal of the pair to leave the corridor and as a “following” for the other one.

To enable inferences about relationship between these values we plotted them in a circular plot (Figure 3C–D) where each color‐coded node of the network represents a single mouse. The size of the solid circle for each mouse represents the total number of times a given mouse followed another mouse, while the size of the dashed circle represents the total number of times the given mouse was followed by another individual. The thickness of arrows connecting pairs of mice represents the strength of their interaction. The numbers by each mouse (node) represent PageRanks (shown as %), that is, weights of nodes in directed following graphs. From such depiction we can deduct which animals in a given cohort were most socially active and what was their role in the group (animals performing the most followings are likely to be at the top of the social ladder, while mice subjected to most followings, that is, the mice with most “leadings” fall lower in that hierarchy). Plots were calculated for every day of the experiment separately allowing for visualization of changes in social dynamics over consecutive days. The dynamics of social interactions of each cohort of BTBR and B6 mice was depicted in a separate figure (for B6 cohort 2 and BTBR cohort 1—Figure 3C–D; for B6 cohort 1 and BTBR cohort 2—Figures [Link], [Link]). To normalize for differences in day‐to‐day activity of each cohort we have provided the same types of graphs drawn for values of following normalized to the total number of followings for the entire cohort on a given day (Figures [Link], [Link]).

The number of followings and leadings for each day of the experiment were also plotted against one another (Figure 3B) to depict the stability of social rank of each individual.

### 2.4 Statistical analysis

Statistical analyses of data were performed with GraphPad Prism6 software, Statistica 13 package and Wolfram Mathematica 12. For social affiliation test, normality of data distributions was assessed with D'Agostino‐Pearson omnibus normality test or Shapiro–Wilk normality test. Between strain and condition (side of the apparatus) comparisons was analyzed using two‐way ANOVA with multiple comparisons. Holm‐Sidak multiple comparison test was used to correct for multiple comparisons. For within group (social vs. non‐social) comparisons in the BTBR strain either paired *t*‐test or Wilcoxon matched‐pairs signed‐rank test were used (depending on data distribution). Pearson correlation was used for analysis of relationship between behavioral measures encoded manually (BehaView) and with EthoVisionXT9.

Analyses of parameters recorded in the Eco‐HAB system were performed primarily with Mann–Whitney test as at least one set of data in each comparison did not pass normality tests mentioned above. Incohort sociability changes were assessed with Kolmogorov–Smirnov test.

For the longitudinal observation study, the correlation between the number of followings and leadings was calculated with Pearson correlation. Spearman correlation coefficient was calculated to compare the probabilities of distributions of ranks of data between cohorts of B6 and BTBR mice.

## 3 RESULTS

### 3.1 Social affiliation test in the three‐chambered apparatus

In search for most reliable parameters describing social affiliation in the three chambered apparatus we attempted to find which parameters recorded manually by trained human observers (with the use of BehaView software) correlated best with parameters measured automatically (with EthoVision XT9, Noldus). To do so we calculated the differences (non‐social – social side of the apparatus) for each parameter from all groups of BTBR mice used in the study and computed Pearson correlation coefficients for each pair of parameters. The resulting correlation matrix (Figure 1B) showed a strong relationship between the two manually scored parameters (time spent on sniffing cups and the number of sniffing bouts, *r*(59) = 0.69, *p* < 0.0001) as well as a strong correlation between the number of manually scored sniffing episodes and the automatically scored distance traveled (*r*(59) = 0.81, *p* < 0.0001). The latter correlated well also with manually scored sniffing time (*r*(59) = 0.52, *p* < 0.001) and the (difference in) time spent in both chambers (*r*(59) = 0.80, *p* < 0.0001, scored automatically). Remarkably, the difference in time spent on either side of the arena (historically the most popular measure of sociability in the three chambered apparatus) correlated poorly with manually scored sniffing time (*r*(59) = 0.37; *p* = 0.006). We thus chose to primarily present data on manually scored sniffing time (Figure 1C–E) but at the same time show automatically scored time in each chamber (Figure 1F) side by side.

Bearing in mind that both housing, handling, and testing conditions may affect the outcome of sociability tests^15^, ^31^ we first tested for differences between B6 and BTBR strains in conditions that fall on two different sides of the stressfulness spectrum. In bright light conditions ANOVA performed on time spent sniffing yielded significant effects of strain (*F*(1, 40) = 31.00, *p* < 0.0001) and side (social vs. non‐social side, *F*(1, 40) = 34.41, *p* < 0.0001) of the apparatus as well as a significant interaction of the two factors (*F*(1, 40) = 11.42, *p* < 0.0016). Holm‐Sidak multiple comparison test showed that B6 males spent significantly more time sniffing the social object than the inanimate object (*p* < 0.0001) and that BTBR mice showed no such preference (*p* = 0.2023, Figure 1C). The amount of time spent on social sniffing by B6 males was also greater than that spent by the social stimulus by BTBR males (*p* < 0.0001). The time spent near the inanimate object was similar in both strains (*p* = 0.2424).

In low‐stress condition, after habituation to transportation and handling by the Experimenter, the result of the social affiliation test was dramatically different (Figure 1D). ANOVA yielded significant effects of strain (*F*(1, 40) = 18,31, *p* = 0.0001) and side (*F*(1, 40) = 49,75, *p* < 0.0001), but not interaction between the two (*F*(1, 40) = 1778, *p* = 0.1899). Both B6 and BTBR males display preference towards the social stimulus (*p* = 0.0005 and *p* < 0.0001). Social approach in the BTBR strain improved upon habituation to a point of exceeding approach displayed to either social (*p* = 0.0009) or non‐social stimuli (*p* < 0.0001) by B6 males.

We then tested other stress‐lowering manipulations for their effectiveness in improving social affiliation in the BTBR strain. We employed: habituation to transportation alone, habituation to transport and handling, exposure to enriched environment, exposure to enriched environment followed by habituation to transportation and handling by the Experimenter and compared these conditions to testing naïve mice in dim light conditions (Figure 1E–F). We found that for manually scored sniffing time (which we believe is the most accurate way of measuring interest in the social stimulus, Figure 1E) naïve mice did not show a preference to social stimulus (*p* = 0.0824), while mice in all groups that underwent manipulation did so (transport *p* < 0.0001; full habituation *p* = 0.0042; enriched environment *p* = 0.0064; enriched environment + habituation *p* = 0.0039). In automatically scored time spent on social side of the apparatus (Figure 1F), this effect was statistically significant only for the habituation to transport group (*p* < 0.0014). Similar results were obtained for automatically scored distance traveled (habituation to transport *p* < 0.04, Table S1). Frequency of sniffing and visits to social side of the apparatus also varied depending on the mode of analysis. For manually scored number of sniffing bouts increased activity was observed after habituation to transport (*p* < 0.0002) and full habituation (*p* < 0.0008) while the number of visits to both side of the apparatus was similar in all groups when automatic measure was applied. All mean ± SEM values for all parameters are presented in Table S2.

To better understand what drove the asocial behavior of naïve BTBR mice in dim light condition we manually scored cage exploration and self‐grooming in that group. We found that the number of episodes and time spent on exploring either side of the apparatus were equal. The primary localization of self‐grooming, on the other hand was in the non‐social (6 out of 10 mice) and central (5 out of 10 mice) parts of the apparatus, while only 4 out of 10 mice displayed self‐grooming on the social side of the apparatus. The average duration of grooming episodes (including that of animals which did not display grooming in given parts of the arena) was also shorter on the social side (28.53 ± 15.23 s) than in the non‐social (62.09 ± 26.56 s) or central (64.24 ± 27.96 s) parts of the arena. The duration of grooming among the animals which did display grooming in a given location was as follows: social side (71.33 ± 26.89 s), non‐social side (97.57 ± 35.48 s) and central part (128.48 ± 38.13 s).

### 3.2 Approach to social scent in the Eco‐HAB system

Eco‐HAB is a semi‐natural environment designed to record the activity of group‐housed mice over prolonged periods of time with no human intervention. As such, it should, after the initial commotion related to introduction to a novel environment and re‐establishing of dominance structure,^32^ lead to diminished stress in the colony.

Here we allowed 3 days of habituation to the novel environment before introducing a social scent in the form of a soiled bedding from a group of unfamiliar, age‐matched male B6 mice. The response to this stimulus was measured as a change in approach ratio. To look at the dynamics of that response we calculated the ratio for three time bins (first hour, first 4 h and the entire 12 h of the dark phase following the introduction of the scent). We found that, similarly to the social affiliation test in the three chambered apparatus, low stress conditions led BTBR mice to display interest in the social stimulus upon its deposition (1 h bin, B6 *p* = 0.0118 as compared with no change in ratio, BTBR *p* = 0.0340, Figure 2B). Interestingly, while in B6 mice social interest was observed during the 1st hour, it disappeared when plotted together with data from the following 3 and 11 h (4 h *p* = 0.9705, Figure 2C and 12 h time bins *p* = 0.3223, Figure 2D). BTBR mice, on the other hand, remained interested in the scent throughout the entire dark phase (4 h bin *p* = 0.0010 and 12 h *p* = 0.0002 time bins, Figure 2C–D). We then decided to test if this was related to their elevated perseverance^33^, ^34^ rather than increased sociability. We looked at the proportion of visits paid, and time spent in the social compartment (as compared with control scent) during first and second half of the dark phase. BTBR mice displayed a different pattern of persistence in the time spent near social scent than B6 mice (*p* = 0.009, Figure 2E) but not in the number of visits (Figure 2F). We then looked at incohort sociability, a parameter developed to assess sociability in pairs of mice.^19^ It allows for assessment of time mice voluntarily spend together and whether that measure changes upon introduction of a social stimulus. Here we have plotted separate plots for B6 and BTBR mice to look at the changes in their sociability upon introduction of the social scent. The graphs represent their activity during the entire dark phase following the introduction of the stimulus. In B6 mice there was no shift in incohort sociability curve in response to exposure to the social scent (Figure 2G). In BTBR mice, on the other hand, we observed a shift towards lower values of incohort sociability in the same situation (Figure 2I). Interestingly, prior to that exposure mean incohort sociability was higher in BTBR mice (*p* = 0.0004) than in B6 mice, but upon exposure the difference disappeared (*p* = 0.3033).

### 3.3 Social network dynamic in the Eco‐HAB system

We looked at 10 days of uninterrupted activity of B6 and BTBR mice (two cohorts/each strain). To illustrate dynamic changes in interactions, for each dark phase (the activity and thus the number of interactions of animals during light phase was low) we plotted all interactions between pairs of mice in a given cohort into circular plots, in which the strength of the interaction was depicted with thickness of the connecting arrows while the number of times each animal was followed or was a leader were represented by the size of nodes of the network (solid circle for followings and dashed for leadings).

By looking at 10 consecutive days of B6 cohort activity we saw that after the initial burst of activity in the new environment, mice settle to a quite stable dominance structure (Figures 3C and S1) with a dominant male performing the most followings (as showed by Winiarski et al.,^30^ here the male marked in red on Figure 3C and yellow on Figure S1), one or two sub‐dominants challenging the dominant, while the remainder of the group settles as subordinates. Despite (or perhaps thanks to) the established structure, the number of interactions between pairs of mice was relatively equal and the size of network nodes and their weight (PageRanks) were stable for all mice (best displayed by the B6 cohort 1, Figure S1).

In BTBR mice we saw a less stable structure. The activity of mice would increase in bouts (in cohort 1 every third day, Figure 3D, in cohort 2 less regularly, Figure S2) especially among a subgroup of four animals (in each cohort). In cohort 1 on “socially active” days the differences in PageRanks were 3–4 fold between the “active” group and the remainder of the cohort. They also changed two‐fold for those “active” animals between “socially active” and “socially inactive” days. In cohort 2 the differences between “active” and “passive” individuals were even larger. To control for these day‐to‐day differences in the number of interactions we normalized the number of followings/leadings to total number of these episodes in the entire cohort on a given day (Figures [Link], [Link]). This manipulation reduced the differences in the network related to increased activity but did not change the size of the “active” subgroup in the BTBR cohorts.

These results suggest that the stability of social structure is what differentiates the two strains. On any given day all B6 mice take active part in forming the social structure, while in BTBR mice only a very limited group of individuals interacts, while others remain passive. This is most likely not related to aggressive encounters (we found that BTBR mice do not engage in fights or push‐backs while in the tube test and that their dominance structure during exposure to food reward is flat, data not shown). The stability of such hierarchy in time is also strikingly different. In BTBR mice it is related to the number of interactions taking place on a given day, while in B6 mice it is stable and independent of such activity.

We also looked at whether the number of leadings and followings performed by each animal is stable over time, similar in both cohorts for each strain and whether there is a relationship between these parameters. We plotted these values against one another (Figure 3B) and found dramatically different distributions of data points for B6 and BTBR mice. In both B6 cohorts data points for given animals were placed in close proximity and the entire data set formed a much denser cluster than in the case of BTBR mice. There was no clear correlation between the number of leadings and followings in B6 mice either. In BTBR mice, on the contrary, data points for most individuals were scattered along an axis and their ranks were highly correlated (cohort1 *r* = 0.948, *p* < 10^−32^; cohort2 *r* = 0.805, *p* < 10^−22^). Comparison of rank distributions between cohorts confirmed these differences. This is in line with the differences in social dominance structure stability depicted in Figures 3 and [Link], [Link]. The position of individual B6 mice in their respective cohorts was stable over the entire length of the experiment, while in BTBR mice even the position within socially active groups fluctuated (e.g., a male marked in blue in Figure 3D tended to be dominant on non‐social days, but lost that position during socially‐active days). In BTBR cohort the position of dominant (marked in green) and submissive (marked in blue) are relatively stable but the position of 2 sub‐dominants marked in orange and turquoise tends to change from day to day.

## 4 DISCUSSION

The data presented here confirms the need for careful selection of behavioral paradigms used to phenotype animal models of neurodevelopmental disorders. The unification of testing conditions in such paradigms is of utmost importance whenever specific units of analysis are to be employed to answer questions regarding criteria and domains within the RDoC network. In this paper we showed that the gold standard for testing social affiliation (a criterion within Systems for Social Processes Domain), the three‐chambered apparatus test, is sensitive to environmental factors affecting stress level in mice. These factors, such as light in the testing room (bright vs. dim), prior exposure to the Experimenter (handling) and transportation as well as housing conditions (enriched environment) can dramatically affect the outcome of the test, suggesting that presentation of social affiliation may depend on the coping strategy adopted by the given strain of mice in stressful novel environments. Our observation explains, at least partially, the between‐laboratory discrepancies in social affiliation test results.^35^, ^36^ Apart from unifying and refining (3R) testing conditions so that they bring out the innate behavioral traits of the mice, emphasis needs to be placed on the choice of parameters reported as measures of social affiliation. We showed here that manually scored sniffing time and frequency (describing direct interactions with the stimulus mouse) correlate well with one another, while only two of the automatically scored measures (distance traveled and time spent on the social side of the apparatus) correlated with manually scored data. In the past, other measures, such as the frequency of visits to the social side of the apparatus were used as a measure of social affiliation^37^, ^38^ although the importance of manual scoring of direct interactions was recognized early on.^12^, ^39^, ^40^ In our hands this parameter did not correlate with either manually scored data or automatically scored distance traveled and time spent on the social side of the apparatus.

When validating a mouse model characterized by social impairments one needs to make sure that the test used for assessment of the deficit is robust and replicable. Otherwise, any effect of intervention, be it behavioral, pharmacological, or surgical, may be lost because of technical issues rather than the nature of manipulation itself. The BTBR mouse strain is one of the most popular subjects to all sorts of interventions (for review see References 41, 42) aiming at improvement of social behavior. To name a few: maternal strain,^43^ home‐cage group composition,^44^ and conventional versus reverse light cycles^45^ were previously tested for their effect on social affiliation. Of those, only housing in mixed‐strain groups improved sociability of BTBR mice.^44^ The effect of strain of the stimulus mouse was also examined, but with mixed results. Yang and collaborators^46^ saw no effect while Ryan and co‐workers^47^ showed that given choice between unfamiliar individuals of B6 and BTBR strains as stimuli, both B6 and BTBR males spent more time sniffing the B6 stimulus mouse.

Similarly, the reports on anxiety in the BTBR strain, as well as its effects on sociability are mixed.^48^, ^49^, ^50^, ^51^, ^52^, ^53^ Our study points to the importance of lowering stress level at the time of testing and as such speaks for improvement of standard protocols used for assessment of social affiliation. One of such manipulations is to house mice in enriched environment prior to the test. In our study housing in the IntelliCage system (TSE, Germany) greatly improved social affiliation in the BTBR strain. This is in line with a recent study showing that inclusion of “running wheels, igloos, toys, tunnels, a maze, and nesting material” in a large home cage both reduces anxiety and improves social affiliation in the BTBR strain.^54^ A similar effect was also observed for the Shank3^ΔC/ΔC^ mouse model of ASD.^55^ In this model enriched environment rescued ASD‐like phenotype of the mutant, including the dependence of novel context recognition on activation of the dopaminergic prelimbic‐tail‐of‐striatum pathway. The introduction of familiar objects or bedding during exposure to novel context also reduced engagement deficits in these mice.

Another manipulation which in our hands proved effective was the habituation of animals to both transport and handling by the Experimenter. This is in line with previous reports showing that habituation to handling not only improves human‐mouse interaction but also lowers human evoked anxiety in several mouse strains.^15^

The nature of social affiliation test in the three chambered apparatus is very specific. It is a brief, restricted, dyadic interactions in a novel environment. While such arrangement may model certain types of natural interactions, both in rodents and humans, it does not permit the mouse to make use of a full repertoire of social behaviors, which in the natural environment are displayed in ethologically size‐relevant groups. It also illustrates only two aspects of social behavior: social motivation and withdrawal, classified under Social Affiliation and Attachment criterion in the RDoC Systems for Social Processes Domain. In our opinion, to fully address social behavior impairments in rodent models of neurodevelopmental disorders we need tools which would address different constructs (e.g., Social Affiliation and Attachment, Perception and understanding of others and Social Communication) of that domain and at the same time allow for reduction of variability/stressors and human interference during testing. To attempt that, more longitudinal strategies and more naturalistic environment are needed.

First attempts at using semi‐natural environment for testing social behaviors in groups of laboratory rodents were performed by McClintock and Adler^56^ to study sexual behavior in rats. The visible burrow system of their design was then used at the Blanchard laboratory^57^, ^58^, ^59^ and the Sakai laboratory (for review see References 21, 60) and then scaled down to house groups of unfamiliar mice.^23^, ^61^ Later it was modified to fit more animals and compartments^22^ and allow for automatic scoring of social behaviors with the use of combined video and RFID tag recording.^32^, ^62^ The latter approach has been used by other groups to track social interactions of mice in complex open spaces^29^, ^63^, ^64^ and in home cage environment.^27^, ^65^ Alternatively, color tracking^66^ and symbol tracking^25^, ^26^ were employed to identify individual mice. These types of recordings allow for observation of voluntary social interactions of groups of mice (usually *n* = 4–10) over the period of several days. Systems employing video only allow for detailed monitoring of interactions between animals in open spaces but fail when visibility is obstructed (in more complex environments). Systems using RFID tagging together with video often restrict the area of analysis to open spaces covered by the RFID antenna matrix and because of heavy load of data tend to be shorter (although Peleh et al.^32^ scored continuous 7 days of recording). For longitudinal studies, we chose to reduce the amount of detail regarding close‐up interactions of individual animals in order to allow for better mapping of social dynamics in large groups of mice (*n* = 10–12), based on their activity in a complex environment composed of four cages. Two of them contain food hoppers and water access (which also provide shelter for mice) while in the other two cages external social stimuli can be presented behind partition (thus mimicking the three chambered apparatus). The Eco‐HAB system was designed to study social approach to social olfactory stimuli^19^ and can be used to present scents from naïve mice (as presented here) or emotionally charged ones^30^ (preprint). Our data show, that after 3 days of habituation both B6 and BTBR mice show an increase in approach when confronted with a social scent introduced (behind partition) to one of the cages at the beginning of fourth dark phase. That interest diminished in B6 mice with time, while in BTBR mice it remained elevated throughout the entire dark phase. This could indicate a lack of habituation to the novel social scent in the latter strain. To test that we looked at persistence, a parameter measured within the dark phase immediately following the introduction of the social scent. Indeed, we found that the number of visits and time spent by BTBR mice near the stimulus were similar throughout the dark phase (not significantly different from 1, which indicates equal values for first and second half of that period). A similar dynamic was observed for the majority of B6 mice, but surprisingly, a subset of them spent more time near the stimulus in the second half of the phase, which given the unchanged number of visits suggests, that they displayed stronger interest in the social stimulus as the dark phase progressed. Such behavior was previously reported for dominant and sub‐dominant males in the social affiliation test.^67^ No such social stratification in persistence was observed in the BTBR strain, which is a phenomenon we later discuss in the light of our longitudinal observation of social network. Another indication of altered responsivity to social stimuli in BTBR mice was observed for the time they voluntarily actively spent together after the introduction of the social scent (at the beginning of the dark phase). The reduction in incohort sociability in this situation indicates that the novel social stimulus caused an increase in individual stimulus exploration, rather than a group coordinated effort to investigate the source of the scent. In B6 mice no such change was observed (for novel social smell). Interestingly, baseline incohort sociability was higher in BTBR mice than in B6 mice, which may seem contrary to the findings of Pobbe et al.,^23^ who reported that BTBR spent less time together (huddling, in both dark and light phases) than B6 mice. This difference has two main reasons. One is that the parameter reported by Pobbe et al. was a direct assessment of huddling behavior (displayed during periods of inactivity), while incohort sociability is a parameter related to activity (it looks at excess time spent together in each compartment of the Eco‐HAB above the time the mice would spend there because of independent exploration of the apparatus). As a value normalized for locomotor activity (which is generally higher in the BTBR strain^52^, ^68^), in periods of immobility it is close to zero, so it best describes changes in behavior during periods of activity and as such cannot be compared with huddling behavior. Another, more biological reason for the discrepancy may lay in the familiarity of the individuals in the cohort. Mice used in our study were cage mates for at least a week prior to the onset of testing, while mice in the Blanchard laboratory were unfamiliar to one another.

One could argue that the source of the scent might also bias the social response of mice tested here. We used soiled bedding from a cage of unfamiliar B6 males as a stimulus for both B6 and BTBR mice. For BTBR mice a scent of B6 mice is a novel stimulus, while for the B6 mice a scent of other individuals of its own (inbred) strain may not represent social novelty. Recent literature suggests, however, that B6 male mice emit as diverse major urinary proteins (MUPs) as outbred strains of mice.^69^ The vomeronasal organ (and its projection neurons in the accessory olfactory bulb) of B6 mice reacts strongly (and with certain degree of individual variability) to the scent of either B6 or BALB/c mice suggesting that chemosensory recognition of individuals from either strain is conserved in inbred mouse strains and encoded by the plastic changes in the activity of accessory olfactory bulb neurons.^70^ Previous reports also show that males from BTBR strain respond with the same amount of scent marking to male urine from either B6 mice or their own strain^71^ so we feel that the social scent used here was an adequate one.

The activity of mice in the Eco‐HAB system is completely voluntary. A fraction of it is solitary, but with 10–12 individuals per cohort most of the time animals interact with one another. The dynamics of these interactions (here represented by followings and their mirroring behavior—leadings) changes over time in large groups of mice. In earlier works using the VBS two main types of social networks were described: ones with despotic dominants and ones with fluctuating social ranks.^72^ In our 10‐day observation we saw, what could be described more as stable versus fluctuating social hierarchy. The first one was presented by B6 mice, which after a short period of initial exploration of the novel environment settled into a quite stable social network. The two B6 cohorts we tested had different profiles, one had a clear social dominance structure (and a very active dominant male) while the other was more “egalitarian.” In both, however, the same animals remained dominant and sub‐dominant (individuals performing the most followings) throughout the entire recorded period. All animals contributed equally to the network of social interactions (as showed by % PageRanks depicting the weight of their nodes in the graph).

In BTBR mice the fluctuation in social interaction was much more striking. In both cohorts there was a subgroup of 4 males, which would periodically (in the first cohort every 3 days, in the second cohort less regularly) interact, mainly with one another. The remainder of the group seemed to passively cope with this outburst of interactions. The position within that group would also change on daily basis (more profoundly in the first cohort, where dominance would change between socially active and inactive days; in the second cohort the changes in social hierarchy were limited to sub‐dominant males).

To test whether these fluctuations are not just a function of outburst of locomotor activity, we normalized the values to the total number of followings in the cohort on a given day. The resulting graphs for B6 cohorts looked almost identical to the raw data ones, while in BTBR cohorts the huge day‐to‐day fluctuations were minimized, but the interactions remained strongest between those limited groups of four individuals per cohort. This effect is most probably not related to aggressive behavior in BTBR cohorts. Our unpublished observations from u‐tube test showed that BTBR mice when confronted with a conspecific in a tube, turn away from one another (and selfgroom) rather than fight or try to push each other out of the tube. This is in line with observations of BTBR mouse behavior in social proximity test.^49^

Since this was an unexpected difference, we looked whether there are differences in correlation of the two forms of interaction recorded in the Eco‐HAB: the leading and the following, between cohorts of B6 and BTBR mice. The distribution of these interactions was strikingly different. While for B6 cohorts it was a tight cluster (especially for cohort1), with points for all days falling near one another for each mouse, in the BTBR mice the points for each mouse for different days would spread along the correlation axis, rather than forming a cluster. The correlation between leading and following was also much stronger in the BTBR strain, suggesting that it was driven by the same individuals. The explanation for this type of instability in the BTBR strain may again come for studying the relationship between social interactions and stress.

While much attention was given to examining the effects of solitude in mice and how stressful such rearing is,^73^, ^74^ it is important to recognize that living in a social group can also be a source of stress (for in‐depth review please see Reference 18) with neuronal, endocrine and immune consequences,^16^, ^31^ which are not equal for each member of the group.^75^, ^76^ Dominant animals often experience stress related to constant challenging of their position by sub‐dominants^72^, ^77^, ^78^ which result with, among others, changes in gene expression in the hippocampus^79^ not observed in subordinates. In the wild, mice display strong territoriality^80^, ^81^ which in laboratory emerges when living spaces are compartmentalized.^82^ Here we did not observe aggression in B6 or BTBR cohorts, but admittedly we did see an increase in such behavior in males of the FVB strain (Nikolaev et al. in prep). This is in line with previous reports on the effect of strain on inter‐male aggression in mice.^83^, ^84^, ^85^

## 5 CONCLUSIONS

Taken together, data presented here show that phenotyping and validation of animal models of neurodevelopmental disorders intended for addressing specific constructs described in the RDoC framework requires careful consideration of behavioral units of analysis (tests) used. While we recognize that both strategies tested here have their good sides (to name the most important: huge database of previous results from other animal models for the social affiliation test and ethologically relevant setting for the Eco‐HAB) and drawbacks (human intervention and bias in the former and the lack of precision in identifying specific types of interactions in the latter), we should strive to develop and optimize their protocols (e.g., by minimizing stress and/or employing machine learning for behavior analysis, especially should Eco‐HAB data be combined with continuous video tracking) in order to increase the preclinical value of our results. The more our methods are guided by the natural ecology of rodent species the better the chance that they will pick up differences relevant to the functioning of our model animal in its' natural environment. By doing so and limiting stress and human intervention during testing we will allow animals to make use of the full repertoire of social behaviors. This in turn should enable us to measure behaviors falling under a broader spectrum of constructs of the RDoC. While standard dyadic social affiliation tests measure primarily social withdrawal/over‐attachment and social motivation belonging to the Affiliation and Attachment criterion of the Systems for Social Processes Domain, testing of group‐housed mice in a semi‐natural environment allows to add processing of social cues and assessment of the strength of social bond (both belonging to that criterion) to the list. It also permits to study emotional contagion (criterion: Perception and understanding of others) and engagement in reciprocal interaction (falling under Social Communication criterion) at the same time. Seeing how many therapeutic strategies and interventions often alter more than one of these aspects, it is crucial that we test as broadly as possible within boundaries set by 3R limitations.

## CONFLICT OF INTEREST

The authors declare no potential conflict of interest.

## Supporting information


**Figure S1** Visualization of social network dynamics in the B6 cohort no.1 (*n* = 13) over 10 consecutive days. Graphs depict the strength of interaction between pairs of animals in each cohort: the size of the solid circle represents the number of times a mouse followed other mice, the size of the dashed circle represents the number of times the given mouse was followed by another individual. The thickness of arrows connecting pairs of mice represents the strength of their interaction. The color‐matched numbers by each node represent PageRanks (shown as %) i.e., weights of nodes in directed following graphs.

**Figure S2** Visualization of social network dynamics in the BTBR cohort no.2 (*n* = 10) over 10 consecutive days. Graphs depict the strength of interaction between pairs of animals in each cohort: the size of the solid circle represents the number of times a mouse followed other mice, the size of the dashed circle represents the number of times the given mouse was followed by another individual. The thickness of arrows connecting pairs of mice represents the strength of their interaction. The color‐matched numbers by each node represent PageRanks (shown as %) i.e., weights of nodes in directed following graphs

**Figure S3** Visualization of normalized social network dynamics in B6 cohort no.1 (*n* = 13) over 10 consecutive days. Graphs depict the strength of interaction between pairs of animals in each cohort: the size of the solid circle represents the number of times a mouse followed other mice normalized by the total number of followings on a given day, the size of the dashed circle represents the number of times the given mouse was followed by another individual normalized to the total number of such episodes on a given day. The thickness of arrows connecting pairs of mice represents the strength (in % of all interactions) of their interaction.

**Figure S4** Visualization of normalized social network dynamics in B6 cohort no.2 (*n* = 10) over 10 consecutive days. Graphs depict the strength of interaction between pairs of animals in each cohort: the size of the solid circle represents the number of times a mouse followed other mice normalized by the total number of followings on a given day, the size of the dashed circle represents the number of times the given mouse was followed by another individual normalized to the total number of such episodes on a given day. The thickness of arrows connecting pairs of mice represents the strength (in % of all interactions) of their interaction.

**Figure S5** Visualization of normalized social network dynamics in BTBR cohort 1 (*n* = 10) over 10 consecutive days. Graphs depict the strength of interaction between pairs of animals in each cohort: the size of the solid circle represents the number of times a mouse followed other mice normalized by the total number of followings on a given day, the size of the dashed circle represents the number of times the given mouse was followed by another individual normalized to the total number of such episodes on a given day. The thickness of arrows connecting pairs of mice represents the strength (in % of all interactions) of their interaction.

**Figure S6** Visualization of normalized social network dynamics in BTBR cohort 2 (*n* = 10) over 10 consecutive days. Graphs depict the strength of interaction between pairs of animals in each cohort: the size of the solid circle represents the number of times a mouse followed other mice normalized by the total number of followings on a given day, the size of the dashed circle represents the number of times the given mouse was followed by another individual normalized to the total number of such episodes on a given day. The thickness of arrows connecting pairs of mice represents the strength of their interaction.

**Table S1** *P*‐values for differences (social vs. non‐social side of the apparatus) measured with either paired t‐test or Wilcoxon matched‐pairs signed‐rank test for BTBR groups tested in dim light conditions (25 lux): naïve (*n* = 10), mice habituated to transportation alone (*n* = 12), mice habituated to both transportation and handling by the Experimenter (*n* = 10), mice previously living in the enriched environment of the Intellicage (TSE, DE) system (*n* = 10), mice previously living in the Intellicage (TSE, DE) system and then habituated to both transportation and handling by the Experimenter (*n* = 9).

**Table S2** Mean ± SEM values for all parameters measured for BTBR groups tested in dim light conditions (25 lux): naïve (*n* = 10), mice habituated to transportation alone (*n* = 12), mice habituated to both transportation and handling by the Experimenter (*n* = 10), mice previously living in the enriched environment of the Intellicage (TSE, DE) system (*n* = 10), mice previously living in the Intellicage (TSE, DE) system and then habituated to both transportation and handling by the Experimenter (*n* = 9).

## Data Availability

All data will be made available upon request. Technical reports for all antennas used throughout the experiments described here are available elsewhere.^30^
