# Supplementary figures and images for: Social deficits in BTBR T+ Itpr3tf/J mice vary with ecological validity of the test
Source: Genes Brain Behav. 2022 May 27;21(5):e12814. doi: 10.1111/gbb.12814 (PMC9744492; doi:10.1111/gbb.12814)

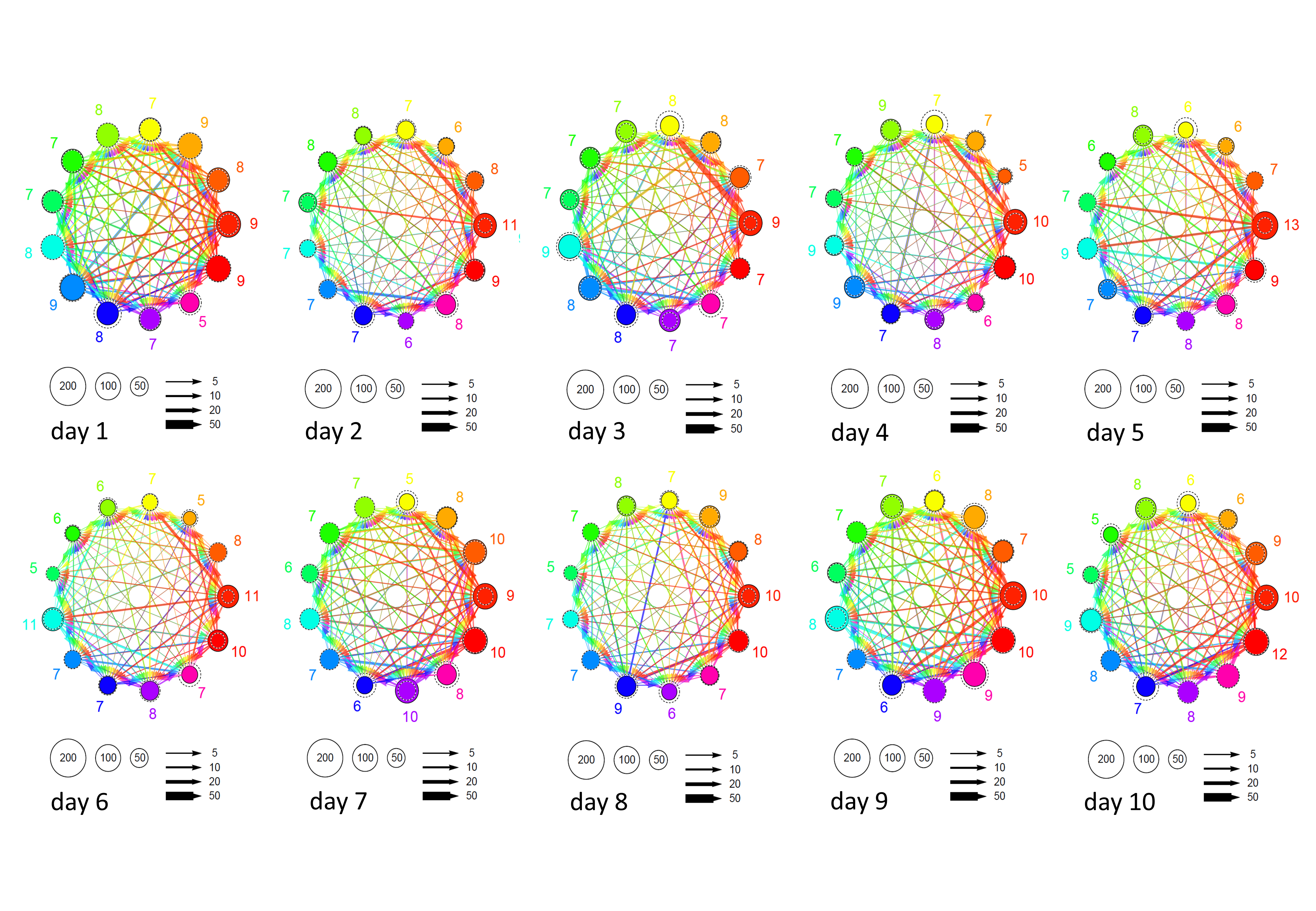

Supplement: Supplementary file 1 — Figure S1 Visualization of social network dynamics in the B6 cohort no.1 (n = 13) over 10 consecutive days. Graphs depict the strength of interaction between pairs of animals in each cohort: the size of the solid circle represents the number of times a mouse followed other mice, the size of the dashed circle represents the number of times the given mouse was followed by another individual. The thickness of arrows connecting pairs of mice represents the strength of their interaction. The color‐matched numbers by each node represent PageRanks (shown as %) i.e., weights of nodes in directed following graphs. [file GBB-21-e12814-s003.tif]

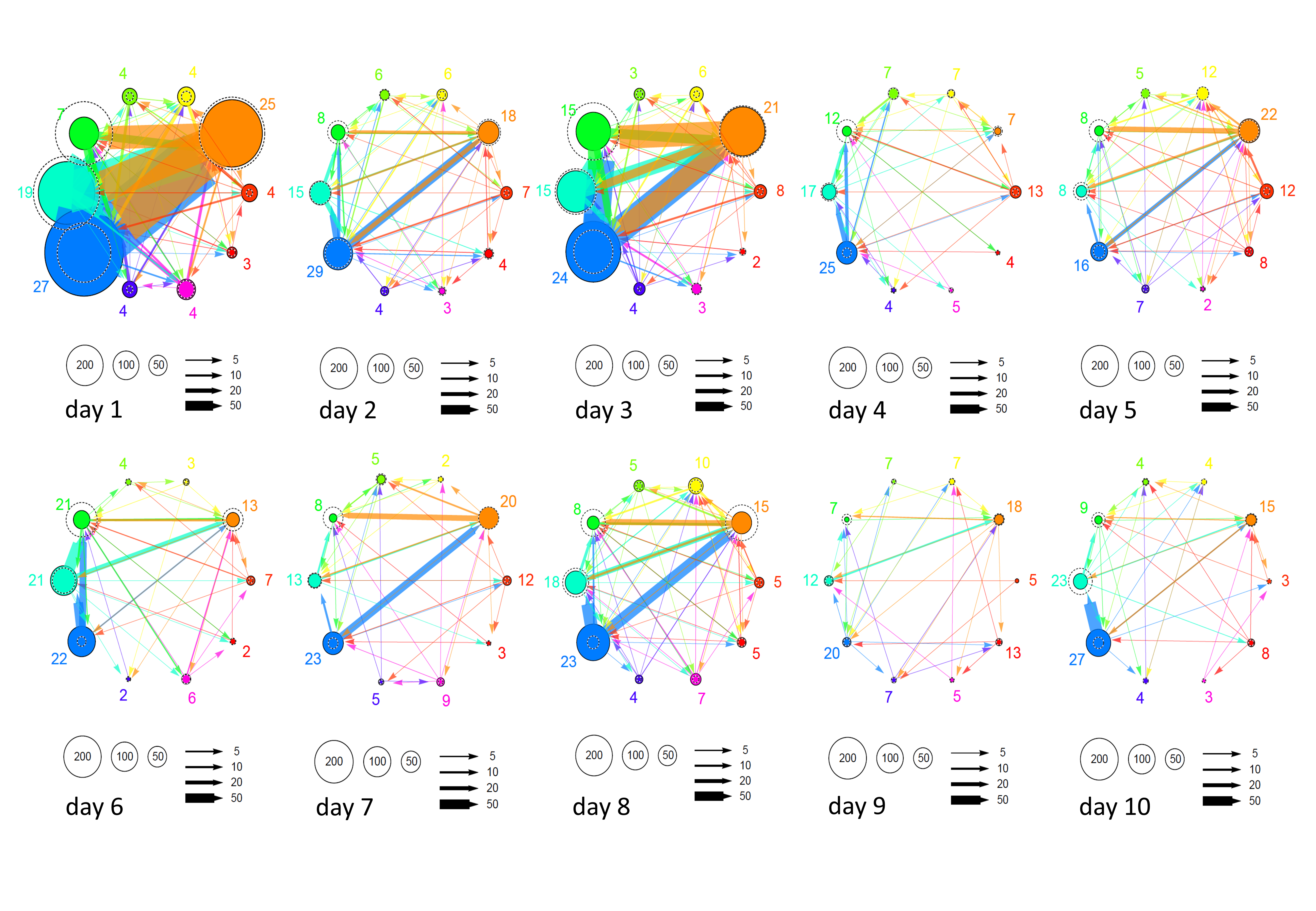

Supplement: Supplementary file 2 — Figure S2 Visualization of social network dynamics in the BTBR cohort no.2 (n = 10) over 10 consecutive days. Graphs depict the strength of interaction between pairs of animals in each cohort: the size of the solid circle represents the number of times a mouse followed other mice, the size of the dashed circle represents the number of times the given mouse was followed by another individual. The thickness of arrows connecting pairs of mice represents the strength of their interaction. The color‐matched numbers by each node represent PageRanks (shown as %) i.e., weights of nodes in directed following graphs [file GBB-21-e12814-s001.tif]

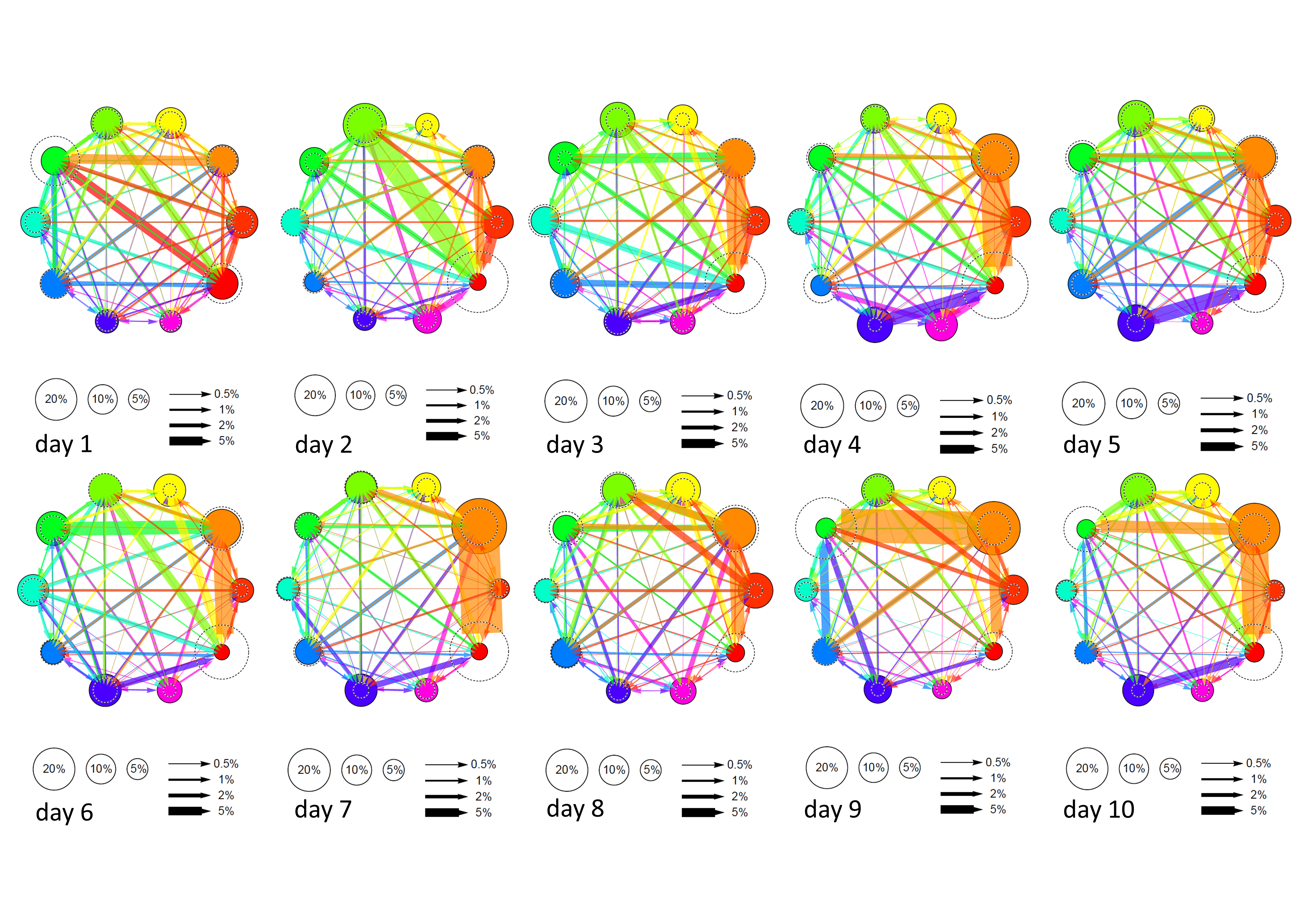

Supplement: Supplementary file 3 — Figure S3 Visualization of normalized social network dynamics in B6 cohort no.1 (n = 13) over 10 consecutive days. Graphs depict the strength of interaction between pairs of animals in each cohort: the size of the solid circle represents the number of times a mouse followed other mice normalized by the total number of followings on a given day, the size of the dashed circle represents the number of times the given mouse was followed by another individual normalized to the total number of such episodes on a given day. The thickness of arrows connecting pairs of mice represents the strength (in % of all interactions) of their interaction. [file GBB-21-e12814-s005.tif]

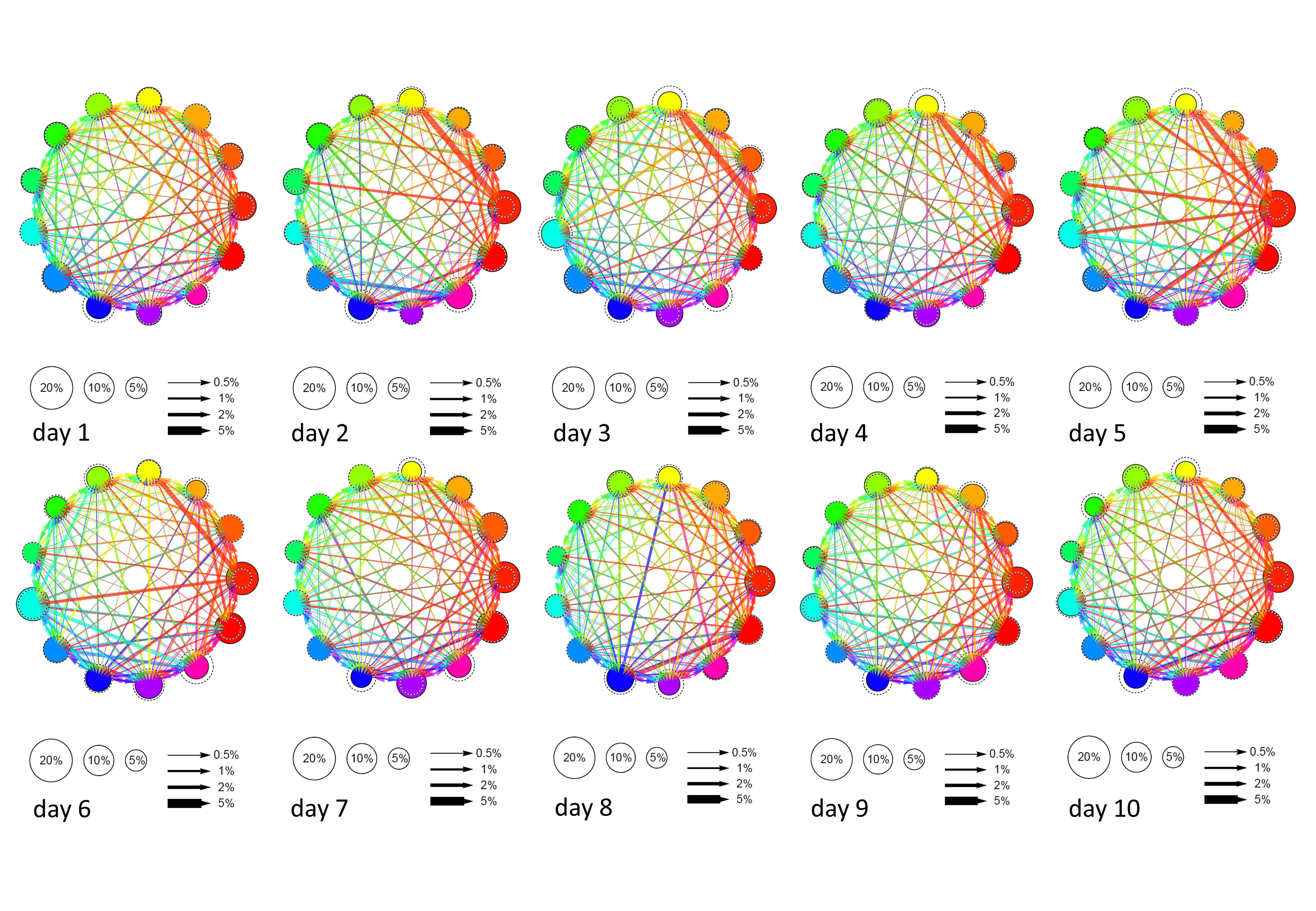

Supplement: Supplementary file 4 — Figure S4 Visualization of normalized social network dynamics in B6 cohort no.2 (n = 10) over 10 consecutive days. Graphs depict the strength of interaction between pairs of animals in each cohort: the size of the solid circle represents the number of times a mouse followed other mice normalized by the total number of followings on a given day, the size of the dashed circle represents the number of times the given mouse was followed by another individual normalized to the total number of such episodes on a given day. The thickness of arrows connecting pairs of mice represents the strength (in % of all interactions) of their interaction. [file GBB-21-e12814-s002.tif]

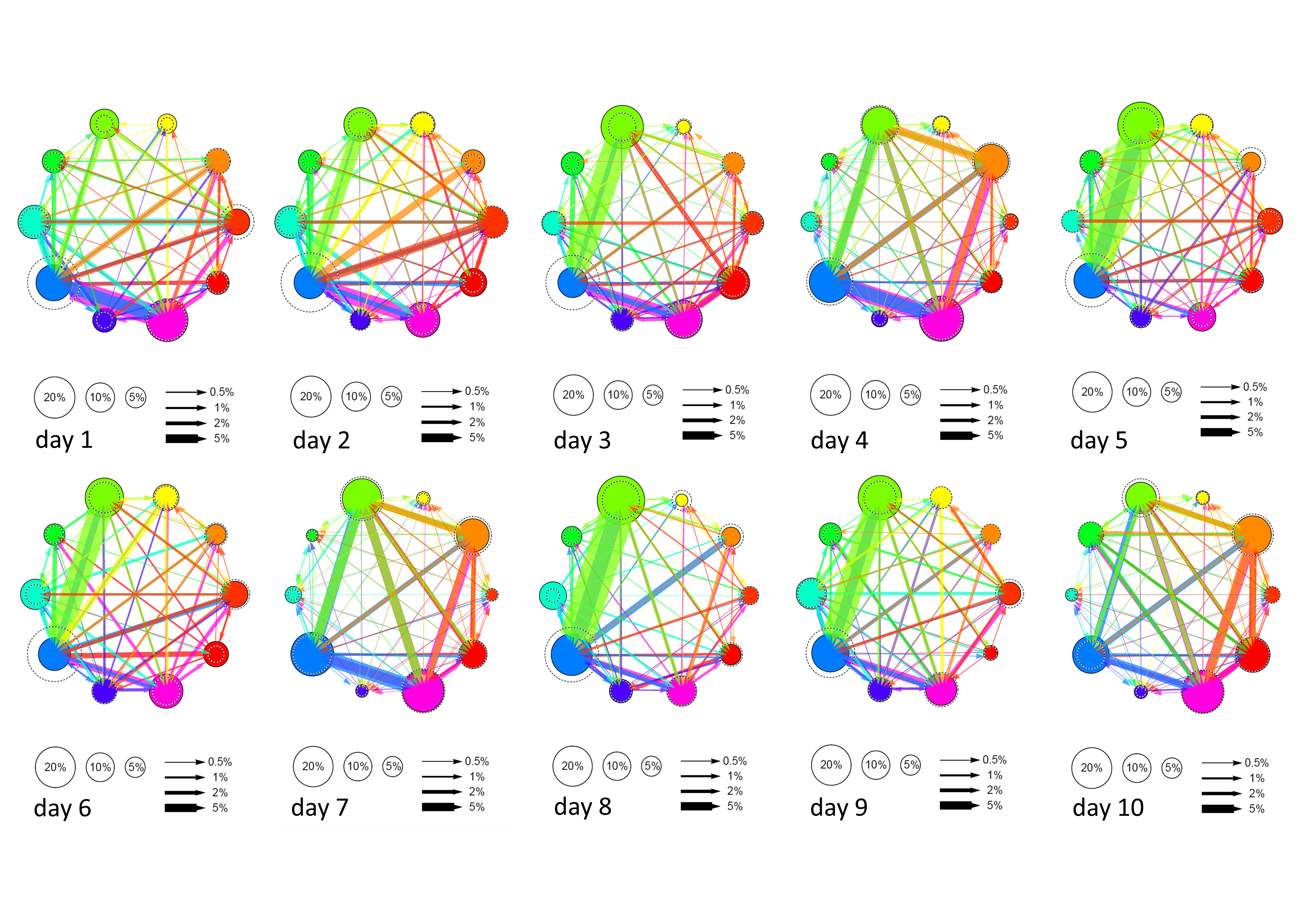

Supplement: Supplementary file 5 — Figure S5 Visualization of normalized social network dynamics in BTBR cohort 1 (n = 10) over 10 consecutive days. Graphs depict the strength of interaction between pairs of animals in each cohort: the size of the solid circle represents the number of times a mouse followed other mice normalized by the total number of followings on a given day, the size of the dashed circle represents the number of times the given mouse was followed by another individual normalized to the total number of such episodes on a given day. The thickness of arrows connecting pairs of mice represents the strength (in % of all interactions) of their interaction. [file GBB-21-e12814-s006.tif]

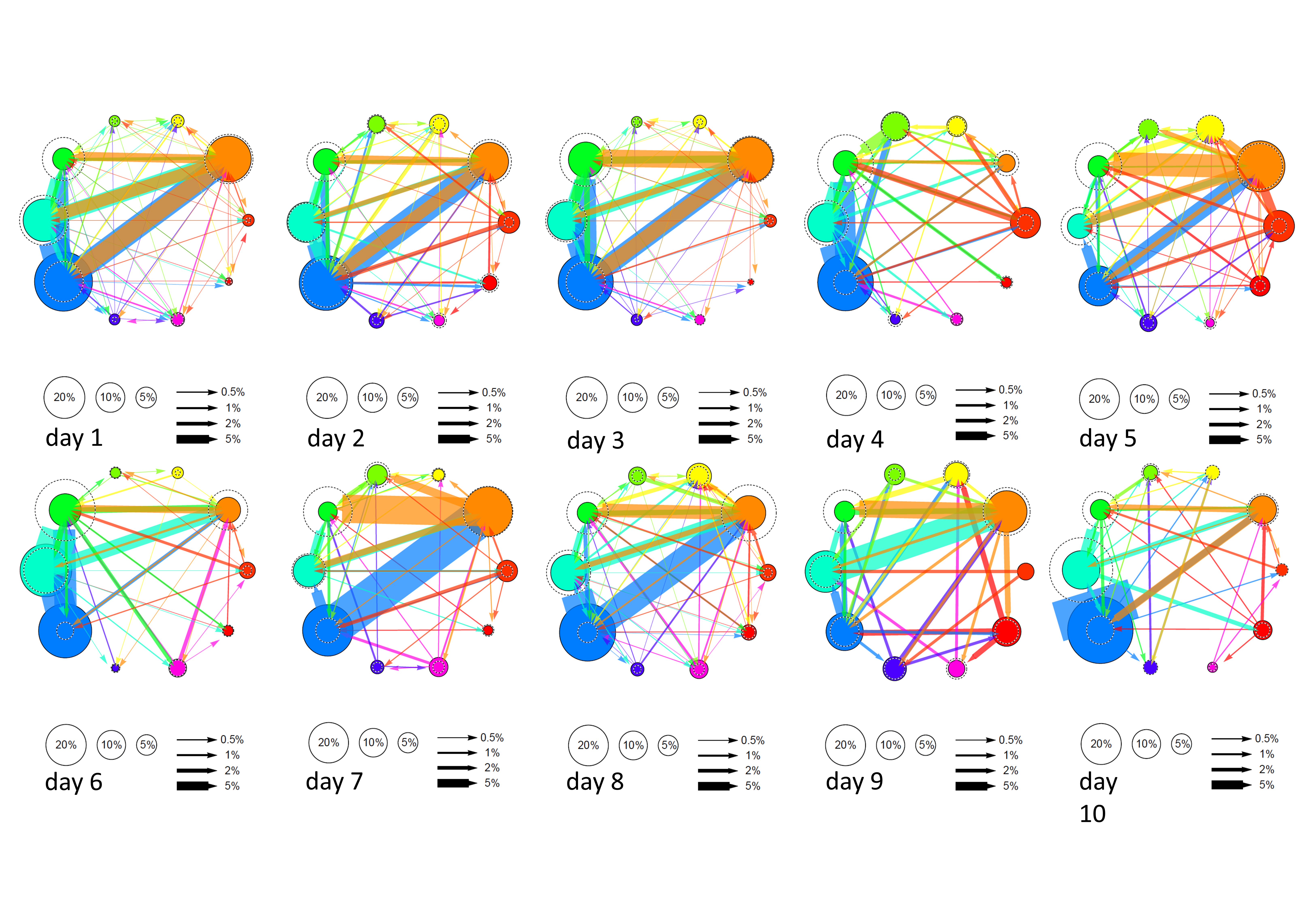

Supplement: Supplementary file 6 — Figure S6 Visualization of normalized social network dynamics in BTBR cohort 2 (n = 10) over 10 consecutive days. Graphs depict the strength of interaction between pairs of animals in each cohort: the size of the solid circle represents the number of times a mouse followed other mice normalized by the total number of followings on a given day, the size of the dashed circle represents the number of times the given mouse was followed by another individual normalized to the total number of such episodes on a given day. The thickness of arrows connecting pairs of mice represents the strength of their interaction. [file GBB-21-e12814-s007.tif]
