# Supplementary material for: Social deficits in BTBR T+ Itpr3tf/J mice vary with ecological validity of the test
Source: Genes Brain Behav. 2022 May 27;21(5):e12814. doi: 10.1111/gbb.12814 (PMC9744492; doi:10.1111/gbb.12814)
Supplement: Supplementary file 7 — Table S1 P‐values for differences (social vs. non‐social side of the apparatus) measured with either paired t‐test or Wilcoxon matched‐pairs signed‐rank test for BTBR groups tested in dim light conditions (25 lux): naïve (n = 10), mice habituated to transportation alone (n = 12), mice habituated to both transportation and handling by the Experimenter (n = 10), mice previously living in the enriched environment of the Intellicage (TSE, DE) system (n = 10), mice previously living in the Intellicage (TSE, DE) system and then habituated to both transportation and handling by the Experimenter (n = 9). [file GBB-21-e12814-s004.docx]

**Table S1** P-values for differences (social vs. non-social side of the apparatus) measured with either paired t-test or Wilcoxon matched-pairs signed-rank test.

|  | Manual scoring | | Automatic scoring | | |
| --- | --- | --- | --- | --- | --- |
|  | Sniffing time | No. sniffing bouts | Time spent | Distance travelled | No. visits |
| Naïve | 0.0645 | 0,3117 | 0,2754 | 0,5520 | 0,1875 |
| Transport | **<0.0001** | **0,0002** | **0,0165** | **0,0065** | **0,0206** |
| Full habituation | **0.0042** | **0,0023** | 0,0864 | 0,1666 | 0,0947 |
| Enriched | **0.0064** | **0,0273** | 0,0836 | 0,1662 | 0,1589 |
| Enriched + hab | **0.0039** | 0,1578 | 0,9096 | 0,6051 | 0,1347 |
