# Supplementary material for: Social deficits in BTBR T+ Itpr3tf/J mice vary with ecological validity of the test
Source: Genes Brain Behav. 2022 May 27;21(5):e12814. doi: 10.1111/gbb.12814 (PMC9744492; doi:10.1111/gbb.12814)
Supplement: Supplementary file 8 — Table S2 Mean ± SEM values for all parameters measured for BTBR groups tested in dim light conditions (25 lux): naïve (n = 10), mice habituated to transportation alone (n = 12), mice habituated to both transportation and handling by the Experimenter (n = 10), mice previously living in the enriched environment of the Intellicage (TSE, DE) system (n = 10), mice previously living in the Intellicage (TSE, DE) system and then habituated to both transportation and handling by the Experimenter (n = 9). [file GBB-21-e12814-s008.docx]

**Table S2** Mean ± SEM values for all parameters measured for Naïve, Transport, Full habituation, Enriched and Enriched + habituation conditions.

|  | Manual scoring | | | | | Automatic scoring | | | | | |
| --- | --- | --- | --- | --- | --- | --- | --- | --- | --- | --- | --- |
|  | Sniffing time [s] | | | No. sniffing bouts | | Time spent [s] | | Distance travelled [cm] | | No. Visits to side | |
|  | soc | | non-soc | soc | non-soc | soc | non-soc | soc | non-soc | soc | non-soc |
| Naïve | | 65.04 ±  7.56 | 43.58 ± 8.88 | 37.30 ± 3.24 | 32.80 ± 3.20 | 228.22 ± 25.77 | 205.05 ± 31.01 | 1219.47 ± 122.23 | 1269.73 ± 119.57 | 14.70 ± 1.92 | 13.70 ± 1.99 |
| Transport | | 118.51 ± 10.91 | 42.10 ± 4.92 | 58.00 ± 3.46 | 37.58 ± 3.11 | 299.72 ± 18.71 | 212.50 ± 14.80 | 1433.50 ± 101.71 | 1161.48 ± 71.77 | 14.92 ± 1.11 | 13.67 ± 0.88 |
| Full habituation | | 96.55 ± 12.03 | 42.42 ± 5.09 | 63.40 ± 4.29 | 40.30 ± 3.84 | 310.51 ± 22.11 | 231.62 ± 19.46 | 1143.24 ± 75.45 | 955.67 ±  95.87 | 12.20 ± 0.70 | 11.30 ± 0.92 |
| Enriched | | 114.97 ± 11.61 | 57.76 ± 6.61 | 68.70 ± 5.81 | 52.70 ± 3.86 | 293.57 ± 14.90 | 237.14 ±  14.97 | 1689.52 ± 121.44 | 1438.29 ±  92.45 | 17.00 ± 0.77 | 16.10 ± 1.07 |
| Enriched + hab | | 78.85 ± 10.28 | 38.78 ± 8.14 | 41.89 ± 6.51 | 33.33 ± 6.18 | 238.81 ± 40.17 | 245.83 ± 43.91 | 934.34 ± 149.88 | 867.95 ±  149.56 | 9.44 ± 1.63 | 8.44 ± 1.58 |
